# Functional Significance of miR-4693-5p in Targeting HIF1α and Its Link to Rheumatoid Arthritis Pathogenesis

**DOI:** 10.3390/ncrna10020022

**Published:** 2024-04-10

**Authors:** Mohd Saquib, Prachi Agnihotri, Ashish Sarkar, Swati Malik, Sonia Mann, Debolina Chakraborty, Lovely Joshi, Rajesh Malhotra, Sagarika Biswas

**Affiliations:** 1 Council of Scientific and Industrial Research (CSIR), Institute of Genomics and Integrative Biology, Delhi University Campus, Mall Road, Delhi 110007, India; saquibmkn@gmail.com (M.S.); prachiagnihotri21@gmail.com (P.A.); ashish2sarkar@gmail.com (A.S.); swatimalik5577@gmail.com (S.M.); mannsonia23@gmail.com (S.M.); debochak55@gmail.com (D.C.); lovelyabhilasha98@gmail.com (L.J.); 2 Academy of Scientific and Innovative Research (AcSIR), Ghaziabad 201002, India; 3 All India Institute of Medical Science (AIIMS), Ansari Nagar, New Delhi 110029, India; rmalhotra62@gmail.com

**Keywords:** miRNA, rheumatoid arthritis, inflammation, apoptosis, synovial fibroblast, SW982

## Abstract

Rheumatoid arthritis (RA) is a chronic inflammatory autoimmune disease that causes joint inflammation and destruction with an unknown origin. Our study aims to elucidate the molecular mechanism behind HIF1α overexpression in RA. Dysregulated miRNA expressions are known to influence gene behavior, thereby enhancing cell proliferation, inflammation, and resistance to apoptosis, contributing to RA development. Our earlier finding indicated that exogenous miRNA similar to miR-4693-5p may modulate RA-related targets. However, the specific role of miR-4693-5p and its targets in RA remain unexplored. In this study, we found that miR-4693-5p was significantly reduced in PBMCs of RA patients, with evidence suggesting it targets the 3′ UTR of HIF1α, thereby potentially contributing to its overexpression in RA. In vitro overexpression of miR-4693-5p leads to the knockdown of HIF1α, resulting in inhibited expression of Survivin to disrupt apoptosis resistance, inflammation suppression, and a reduction in the total cellular ROS response in SW982 and RAFLS cells. The results were validated using the CIA Rat model. In conclusion, this study provides a crucial foundation for understanding the functional role of miR-4693-5p. These findings improve our understanding and provide novel insights into the molecular mechanisms underlying RA pathogenesis.

## 1. Introduction

Rheumatoid arthritis (RA) is a chronic inflammatory autoimmune disease that causes joint destruction with unknown origin. Approximately 1% worldwide and 0.92% of the Indian population are affected by RA [1]. Currently, anti-citrullinated protein antibodies (ACCPA) and rheumatoid factor (RF) are the most widely available non-specific markers, and treatments include DMARDs, NSAIDs, etc., which have severe side effects [2].

Previous reports showed that the high metabolic requirement of proliferative cells and intraarticular pressure generates an oxygen demand, causing hypoxia of the synovial joint. As a result, the prominent protein HIF1α (Hypoxia Inducible Factor 1 Subunit Alpha) gets overexpressed, contributing to RA pathogenesis [3]. However, the molecular mechanism of HIF1α overexpression in RA is unknown. As RA is affected by various factors like cytokines, immune cell activities, joint inflammation, and immune complex accumulation [4], it is necessary to study the possible regulatory molecule that targets HIF1α. Therefore, we focused on knockdown studies of HIF1α to understand the above underlying factors. Recently, the involvement of non-coding RNA (ncRNAs), particularly microRNA (miRNA), regulating the gene expression associated with the disease was reported [5]. It was also reported that HIF1α can be regulated by miRNAs and that miR-210 regulates HIF1α negatively, affecting the dynamic equilibrium of Th17/Treg cells in RA [6]. Another report claimed that pharmacological inhibition of HIF1α by a short hairpin RNA (shRNA) lentiviral expression vector improved the clinical manifestations of RA [7].

As miRNA is a potent gene expression regulator, we speculated that a specific miRNA might be involved in HIF1α regulation. We previously reported that exogenous miRNA similar to miR-4693-5p may modulate RA-related targets [8,9]. The altered miRNA expressions are key controls on gene behavior, contributing to the development of inflammatory conditions [10]. For example, Legg-Calvé-Perthes is an inflammatory disease with high expression of miR-4693-5p that promotes endothelial cell dysfunction and osteoclastogenesis [11]. In another report, miR-4693-5p targeted thyroid hormone receptor-interacting protein 13 (TRIP) 13 to promote apoptosis and suppress cell proliferation [12]. In this study, we found that miR-4693-5p was significantly reduced in the peripheral blood mononuclear cells (PBMCs) of RA patients. This result led us to believe that miR-4693-5p may play a role in the pathogenesis of RA as an endogenous molecule, which has not been reported to date. Therefore, we focused our research on validating the functional role of miR-4693-5p, speculating that miR-4693-5p may be involved in RA pathogenesis. We hypothesized that employing endogenous molecules miR-4693-5p to modulate HIF1α expression may play an important role in preventing RA development. Therefore, in vitro validation of miR-4693-5p using SW982 and rheumatoid arthritis fibroblast-like synoviocytes (RAFLS) cells, followed by in vivo validation using a collagen-induced arthritis (CIA) rat model, was attempted. Overexpression of miR-4693-5p confirms that it regulates HIF1α directly, playing a role in maintaining homeostasis of RA joints by inducing apoptosis, proinflammatory cytokines, and total cellular reactive oxygen species (ROS) suppression. These findings improved our understanding and provided novel insights into the molecular mechanisms driving the pathogenesis of RA.

## 2. Results

### 2.1. miR-4693-5p Expression Decreased in RA PBMCs

**Figure 1:**
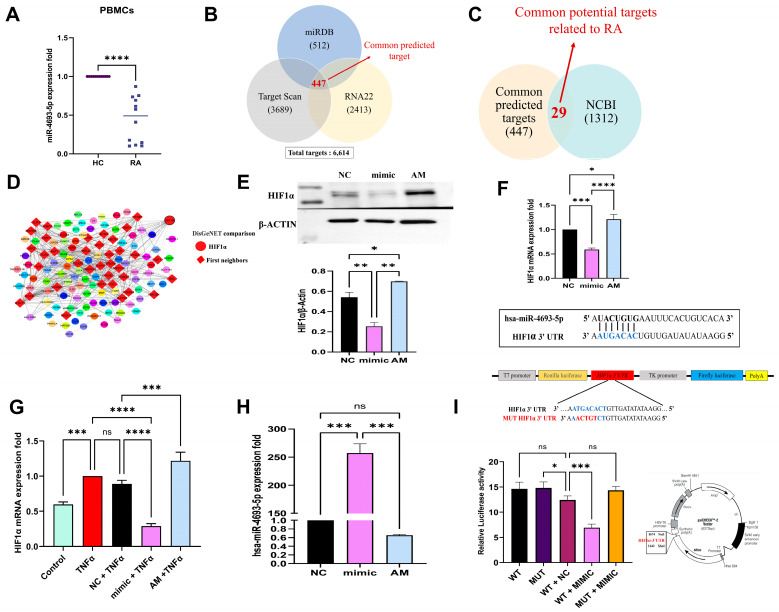
Target identification of miR-4693-5p: (**A**) Downregulated expression level of miR-4693-5p in RA PBMCs compared with Healthy Controls (*n* = 12 each) by stem-loop PCR (**B**) Venn diagram of common 447 predicted targets identified using miRDB (512), TargetScan (3689), and RNA22 (2413). (**C**) Identified 29 common potential targets related to the RA database of NCBI represented by Venn diagram. (**D**) Cytoscape interpretation of first neighbor or directly regulated (in Red) targets of HIF1α. (**E**) Western blot analysis showing significantly decreased HIF1α levels (~2.2 fold) in miR-4693-5p transfected SW982 cells at 25 nM. (**F**) Downregulated HIF1α mRNA expression by qRT-PCR in uninduced and (**G**) TNFα-induced miR-4693-5p-transfected SW982 cells at 25 nM. (**H**) Post-transfection overexpression levels of miR-4693-5p in transfected (25 nM) SW982 cells. (**I**) Dual luciferase assay of human HIF1α 3′ UTR binding sequence of miR-4693-5p significantly downregulated (~2 fold) the luciferase activity in Luc-HIF1α-WT 3′ UTR. (NC: Negative Control, mimic: synthetic miR-4693-5p, AM: AntimiR, GAPDH: Glyceraldehyde 3-phosphate dehydrogenase, TNF-α: Tumor Necrosis Factor-Alpha, HC: Healthy Control, RA: Rheumatoid arthritis, WT: Wild type, MUT: Mutant, level of statistical significance: * = *p* ≤ 0.05, ** = *p* ≤ 0.01, *** = *p* ≤ 0.001, **** = *p* ≤ 0.0001).

PBMCs can imitate conditions in synovial tissue, and their dysregulated miRNAs play important roles in RA pathogenesis and are employed as disease activity indicators [13]. Hence, we checked the expression of miR-4693-5p in PBMCs. We observed that the expression of miR-4693-5p was significantly downregulated in the PBMCs of RA (*n* = 12) patients compared with Healthy Controls (HC, *n* = 12) (Figure 1A), confirming the link between miR-4693-5p and RA.

Next, we explored the regulatory mechanism behind the reasons for the HIF1α overexpression in RA. We considered miR-4693-5p as a potential regulator of HIF1α expression because miRNA is known for its powerful influence on gene expression.

### 2.2. In Silico Target Prediction of miR-4693-5p

Target prediction analysis revealed 447 common targets from the three miRNA prediction databases: miRDB (512), TargetScan (3689), and RNA22 (2413) (Figure 1B) [14]. Targets were matched with RA-related genes acquired from NCBI (1312), yielding 29 RA-specific targets (Figure 1C). HIF1α was selected because of its high total context score, 8mer binding site, and increased expression in RA. The 447 common targets were compared with DisGeNET (RA disease-related targets) [15] for better potential target selection. Therefore, 159 proteins having gene-disease association (GDA) scores of ≥0.1 were identified, and ~1/3rd of the proteins (first neighbors, red color) (Figure 1D) were found to be directly regulated by HIF1α, showing an important regulator in the development of RA.

### 2.3. miR-4693-5p Directly Targets HIF1α

HIF1α is a transcription factor strongly expressed in the intimal lining of synovial tissue and plays a role in cell proliferation, metabolism, angiogenesis, and cell survival in RA [16]. In silico results revealed that miR-4693-5p may target HIF1α. To validate the regulation of HIF1α by miR-4693-5p, dose standardization experiments were performed. WB analysis of HIF1α expression at various 5–50 nm concentrations was conducted (Supplementary Figure S1). Significant downregulation of HIF1α expression at 25 nM was observed; therefore, this concentration was selected for further experiments.

Further, after transfection of miR-4693-5p in SW982 cells, significant downregulation of HIF1α was revealed at 25 nM by WB (~2.2 fold) (Figure 1E) and qRT-PCR (Figure 1F) compared with the Negative Control (NC) and recovered in the presence of miRNA inhibitor (AM). A similar downregulated profile of HIF1α (mimic + TNFα) at mRNA level was also revealed in TNFα-induced SW982 cells compared with TNFα alone and Negative Control (NC) + TNFα (Figure 1G). An overexpressed level of miR-4693-5p was seen at 25 nM in transfected SW982 cells compared with the Negative Control (NC) (Figure 1H). These results indicate that miR-4693-5p significantly downregulates HIF1α expression.

### 2.4. miR-4693-5p Binds to 3′ UTR of HIF1α

To validate that HIF1α is a direct target of miR-4693-5p, a reporter vector containing a luciferase coding gene with predicted specific binding sites of miR-4693-5p was constructed. Wild type and mutated sequence 3′ UTR of HIF1α was constructed (Supplementary Figure S2) to check the regulation of miR-4693-5p. We found that miR-4693-5p significantly downregulates (~2 fold) the luciferase activity in Luc-HIF1α-WT 3′ UTR but not in Luc-HIF1α-MUT 3′ UTR (Figure 1I). Therefore, the results indicate that miR-4693-5p was directly bound to the seed region of HIF1α 3′ UTR, ultimately reducing luciferase activity in the WT + mimic set.

### 2.5. TNFα Induces HIF1α Expression and Establishment of RA

**Figure 2:**
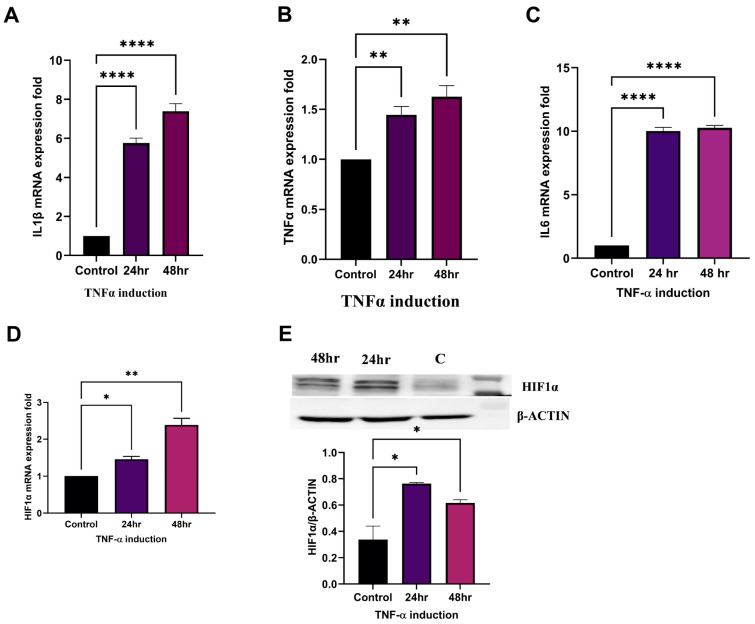
HIF1α expression in TNFα-induced SW982 cells: Increased mRNA level of pro-inflammatory cytokines showing the establishment of inflammatory conditions. (**A**) IL1β, (**B**) TNFα, (**C**) IL6, and (**D**) HIF1α mRNA expressions after induction with TNFα at 10 ng/mL for 24 h and 48 h in SW982 cells by qRT-PCR. (**E**) An increased level of HIF1α (~2 fold) was examined after induction with TNFα at 10 ng/mL for 24 h and 48 h in SW982 cells using Western blot analysis, using β-actin as a loading control. (NC: Negative Control, mimic: synthetic miR-4693-5p, AM: AntimiR, GAPDH: Glyceraldehyde 3-phosphate dehydrogenase, TNF-α: Tumor Necrosis Factor-Alpha, level of statistical significance: * = *p* ≤ 0.05, ** = *p* ≤ 0.01, **** = *p* ≤ 0.0001).

In order to mimic the conditions of RA, we assessed the levels of proinflammatory cytokines in TNFα-induced SW982 cells at concentrations of 10 ng/mL [17] measured at both 24 h and 48 h. The results showed increased mRNA levels of IL1β (Figure 2A), TNFα (Figure 2B), and IL6 (Figure 2C), confirming the establishment of an inflammatory state. These findings support the pivotal role of TNF-α in the pathogenesis of RA. HIF1α helps cells to survive in low-oxygen conditions, potentially speeding up RA development. Surprisingly, even in normal oxygen levels, HIF1α can be triggered by various parameters like thrombin, vasoactive peptides, growth factors, insulin, inflammatory agents (LPS), proinflammatory cytokines (TNF-α, IL-1β), ROS, etc., thereby stabilizing HIF1α protein (32). Our results show that upregulated levels of HIF1α in TNFα induced SW982 cells at 24 and 48 h at both mRNA (Figure 2D) and protein levels compared with the controls (Figure 2E) (~2 fold). The results thus indicate that TNFα induces the expression of HIF1α, contributing to the establishment of an inflammatory condition.

### 2.6. In Silico Target Interaction of HIF1α

**Figure 3:**
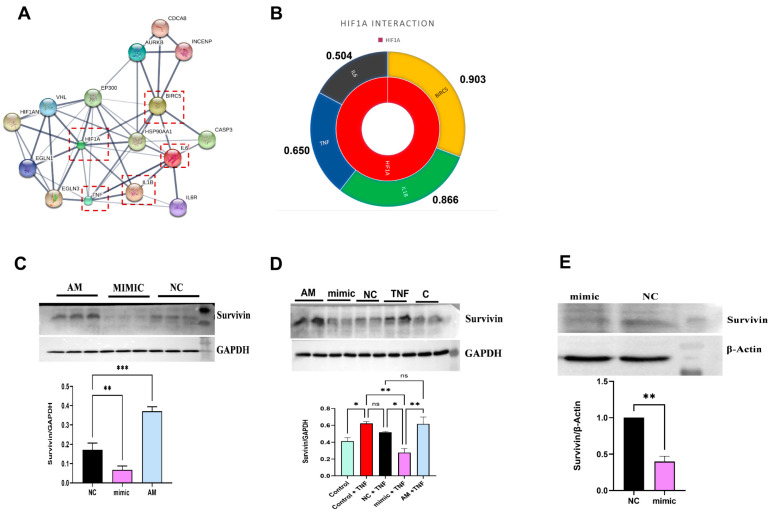
Downstream target analysis and connection with HIF1α: (**A**) STRING network interaction of HIF1α with Survivin (BIRC5) and proinflammatory cytokines IL1β, TNFα, and IL6. (**B**) The interaction score of HIF1α to Survivin is higher (0.903) compared with proinflammatory cytokines IL1B (0.866), TNFα (0.650), and IL6 (0.504). (**C**) Western blot analysis showing downregulated Survivin expression in transfected miR-4693-5p (25 nM) uninduced (~2.6 fold) and (**D**) in TNFα-induced (~1.7 fold) SW982 cells and (**E**) in RAFLS (~2.0 fold) for 48 h. β-actin and GAPDH were used as loading controls. (NC: Negative Control, mimic: synthetic miR-4693-5p, AM: AntimiR, GAPDH: Glyceraldehyde 3-phosphate dehydrogenase, TNF-α: Tumor Necrosis Factor-Alpha, level of statistical significance: * = *p* ≤ 0.05, ** = *p* ≤ 0.01, *** = *p* ≤ 0.001).

The literature suggests that HIF1α is a critical transcriptional regulator of Survivin, facilitating binding to the Survivin promoter directly and stimulating its transcription [18]. Our protein–protein (PPI) interaction (STRING and STITCH databases) result demonstrated a significant role of HIF1α in the pathogenesis of RA by directly interacting with proinflammatory cytokines (TNFα, IL6, IL1β) and Survivin (BIRC5) (Figure 3A). The highest interaction score was observed between HIF1α and Survivin (0.903) compared with proinflammatory cytokines IL1β (0.866), TNFα (0.650), and IL6 (0.504) (Figure 3B), indicating a direct relation of Survivin (downstream target) with HIF1α. Survivin was, therefore, chosen for downstream analysis of miR-4693-5p to emphasize its potential significance in the regulatory mechanisms underlying RA.

### 2.7. miR-4693-5p Reduced the Expression of Survivin, a Downstream Target of HIF1α

In normoxic conditions, HIF1α regulates Survivin expression directly, and in RA, Survivin level is highly upregulated compared with Healthy Controls [18]. Therefore, the expression level of Survivin was checked again in the present study using ELISA of the plasma samples of RA (*n* = 8) and HC (*n* = 8), and an increased expression of Survivin was revealed, indicating its direct relation with disease pathogenesis (Supplementary Figure S3).

Further, significant downregulation of Survivin was revealed after transfection of miR-4693-5p in SW982 cells without (Figure 3C) (~2.6 fold) and with (Figure 3D) (~1.7 fold) TNFα-induction and also in RAFLS compared with the Negative Control (NC) (Figure 3E) (~2 fold). These findings strongly suggest that the knockdown of HIF1α leads to significant inhibition of Survivin expression levels, both in the absence and presence of TNFα induction, in SW982 and RAFLS cells. This reinforces the intricate relationship between HIF1α, Survivin, and the regulatory mechanisms contributing to the pathogenesis of RA.

### 2.8. miR-4693-5p Promotes Apoptosis in RA

**Figure 4:**
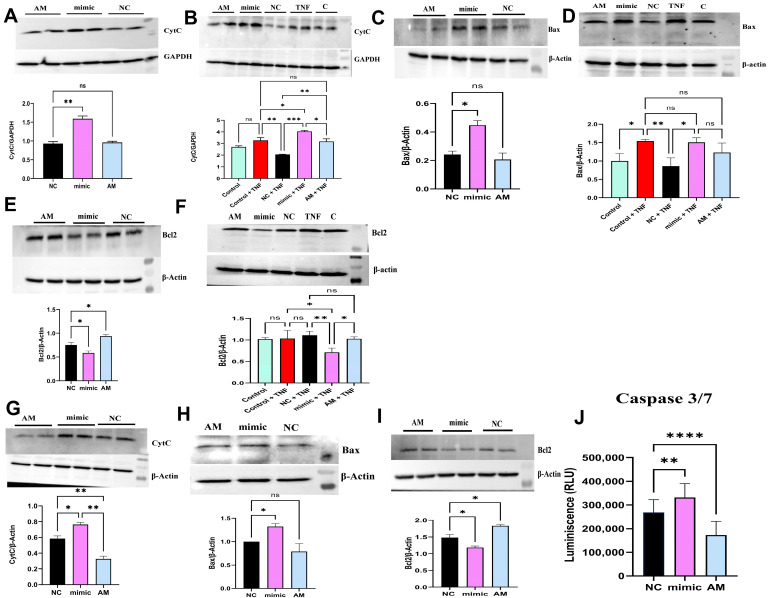
miR-4693-5p promoted cell apoptosis in SW982 and RAFLS cells: Western blot analysis in transfected miR-4693-5p at 25 nM in SW982 and RAFLS cells for 48 h. (**A**) Upregulated CytC expression in uninduced cells (~1.6 fold) and (**B**) TNFα-induced cells (~2 fold). GAPDH was used as the loading control. (**C**) Increased expression of Bax (~2 fold) in uninduced cells and (**D**) in TNFα-induced (~1.5 fold) SW982 cells. β-Actin was used as the loading control. (**E**) Downregulated expression of Bcl2 (~1.3 fold) in uninduced and TNFα-induced (**F**) (~1.5 fold) SW982 cells. β-Actin was used as a loading control. (**G**) Western blot analysis in transfected miR-4693-5p at 25 nM in RAFLS cells for 48 h, showing increased expression of CytC (~1.3 fold) and (**H**) Bax (~1.4 fold). β-Actin was used as the loading control. (**I**) Downregulated expression of Bcl2 (~1.3 fold). β-Actin was used as the loading control. (**J**) Increased Relative Luminescence (~1.2 fold) of caspase 3/7 was measured in RAFLS cells using the Caspase-Glo^®^ 3/7 assay in miR-4693-5p-transfected RAFLS cells. (NC: Negative Control, mimic: synthetic miR-4693-5p, AM: AntimiR, GAPDH: Glyceraldehyde 3-phosphate dehydrogenase, B-Actin: β-Actin, TNF-α: Tumor Necrosis Factor-Alpha, level of statistical significance: * = *p* ≤ 0.05, ** = *p* ≤ 0.01, *** = *p* ≤ 0.001, **** = *p* ≤ 0.0001).

Survivin is an antiapoptotic protein and is overexpressed in RA, inhibiting Cytochrome C’s expression, proapoptotic proteins, and effector caspases [19]. To investigate the impact of miR-4693-5p on apoptosis, Western blot (Cyt C, Bax, and Bcl2) and Caspase-Glo 3/7 assays were attempted. The results showed upregulation of the proapoptotic protein CytC without (Figure 4A) (~1.6 fold) and with (Figure 4B) (~2 fold) TNFα-induced SW982 cells and also upregulation of Bax in TNFα-uninduced (Figure 4C) (~2 fold) and -induced (Figure 4D) (~1.5 fold) SW982 cells, with downregulation of antiapoptotic Bcl2 expression in TNFα-uninduced (Figure 4E) (~1.3 fold) and -induced (Figure 4F) (~1.5 fold) SW982 cells. Similarly, the upregulation of CytC (Figure 4G) (~1.3 fold) and Bax (Figure 4H) (~1.4 fold) and downregulation of Bcl2 (Figure 4I) (~1.3 fold) were also revealed by RAFLS. Further, the upregulation of relative luminescence (~1.2 fold) of caspase 3/7 (Figure 4J) in RAFLS was also demonstrated. These results collectively indicate that miR-4693-5p induces apoptosis stimulation in both SW982 cells and RAFLS, suggesting a potential regulatory role in apoptosis-related pathways.

### 2.9. miR-4693-5p Inhibits Inflammatory Cytokines Production

**Figure 5:**
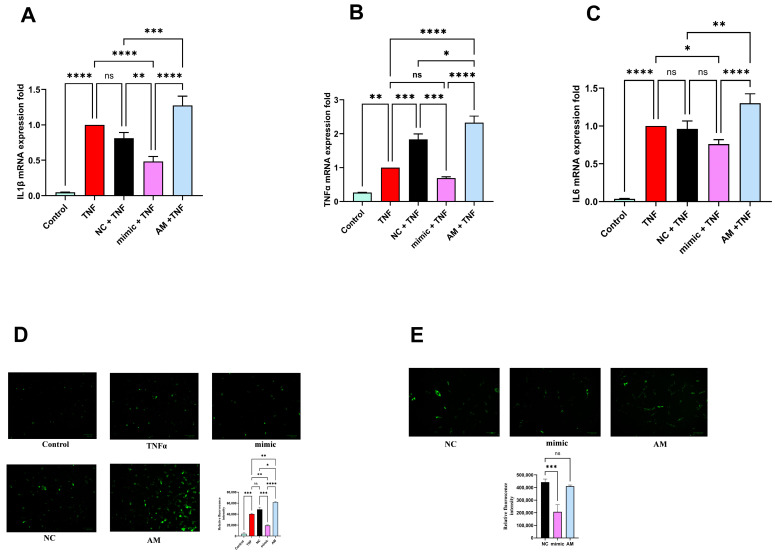
Anti-inflammatory and ROS inhibition effect of miR-4693-5p in SW982 cells and RAFLS: Decreased mRNA level of pro-inflammatory cytokines by qRT-PCR. (**A**) IL1β, (**B**) TNFα, and (**C**) IL6 expressions in SW982 cells transfected with miR-4693-5p after TNFα induction. GAPDH was used as an internal control. Intracellular ROS analyzed in transfected miR-4693-5p in (**D**) TNFα induced SW982 cells and (**E**) RAFLS. miR-4693-5p-inhibited ROS production, measured by relative fluorescence intensity compared with TNFα-induced and NC. DCFDA green fluorescence positive cell population was measured and normalized with a grayscale image. (TNF-α: Tumor Necrosis Factor-Alpha, IL: interleukin, NC: Negative Control, mimic: synthetic miR-4693-5p, AM: AntimiR, GAPDH: Glyceraldehyde 3-phosphate dehydrogenase, level of statistical significance: * = *p* ≤ 0.05, ** = *p* ≤ 0.01, *** = *p* ≤ 0.001, **** = *p* ≤ 0.0001).

The major characteristics of RA joints are inflammation and hypoxia, which activate HIFs to regulate the expression of pro-inflammatory cytokines [16]. A recent report showed that HIF1α increases the production of proinflammatory cytokines like IL-6, IL-8, TNF-α, and IL-1β [20]. Therefore, we evaluated the effect of HIF1α knockdown on proinflammatory cytokines levels and found downregulation of the proinflammatory cytokines IL1β (Figure 5A), TNFα (Figure 5B), and IL6 (Figure 5C) at mRNA level compared with the Negative Control (NC) in transfected TNFα-induced SW982 cells, suggesting that knockdown of HIF1α significantly reduced the expression levels of proinflammatory cytokines. These findings indicate that miR-4693-5p exhibits anti-inflammatory potential by targeting HIF1α.

### 2.10. miR-4693-5p Reduces Cellular ROS Level

RA is also associated with elevated levels of ROS, and maintaining a balance between HIF1α and ROS production is critical to minimize oxidative damage [21]. In this study, we assessed oxidative stress by measuring total intracellular ROS in miR-4693-5p transfected TNFα-induced SW982 cells and RAFLS. Fluorescence signals from DCFDA dye indicated a substantial increase in intracellular ROS production in TNF-α-induced cells compared with untreated control SW982 cells. However, miR-4693-5p transfection inhibited TNF-α-induced intracellular ROS production in SW982 cells (Figure 5D) and RAFLS (Figure 5E). These results suggest the potential of miR-4693-5p in modulating oxidative stress, providing insights into its therapeutic relevance in managing the oxidative aspects of RA.

### 2.11. In Vivo Validation

#### 2.11.1. miR-4693-5p Reduces Macroscopic Arthritic Score and Pro-Inflammatory Cytokines

**Figure 6:**
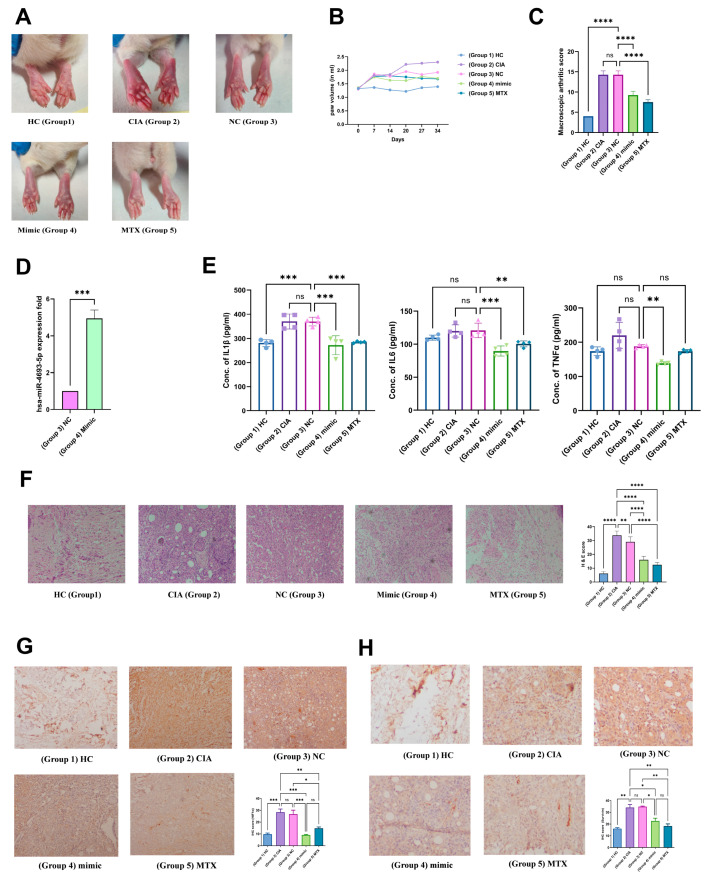
The effect of miR-4693-5p on the Collagen-Induced Arthritis (CIA) Rat model: (**A**) Visual representation of the hind paws of rats from each group, where edema and redness were reduced in Groups 4 and 5 compared with Groups 2 and 3. (**B**) Graphical representation of measured paw volumes from day 0 to day 34, depicting the changes in paw volume in Group 4 compared with Groups 2, 3, and 5. (**C**) The macroscopic arthritic score was measured on the 28th day, and a significant reduction in redness and swelling was observed in Group 4 compared with Groups 2 and 3. (**D**) Fold change of miR-4693-5p in rat synovium by qRT-PCR found significantly upregulated levels of miR-4693-5p in Group 4 compared with Group 3 after normalization with U6 loading control. (**E**) The proinflammatory cytokine levels were measured using quantitative ELISA analysis in rat plasma in Groups 1 to 5, showing the downregulation of IL1 β, IL-6, and TNFα levels in Group 4 compared with Groups 2 and 3. (**F**) The H&E staining shows decreased cell inflammation (pink color) in Groups 4 and 5 compared with Groups 2 and 3. The analysis of cell infiltration in the synovium was measured and found to be downregulated in Groups 4 and 5 compared with Groups 2 and 3. (**G**,**H**) Immunohistochemistry assay for HIF1α and Survivin expression in rat synovium of Group 1 to 5 rats. Typical images of (**G**) HIF1α and (**H**) Survivin expression in synovial tissues showing that HIF1α and Survivin were expressed at low levels in the synovium of Groups 4 and 5 compared with Groups 2 and 3. (Group 1: Healthy or HC, Group 2: CIA, Group 3: CIA + Negative Control (NC), Group 4: CIA + miR-4693-5p mimic, Group 5: CIA + MTX (standard drug), level of statistical significance: * = *p* ≤ 0.05, ** = *p* ≤ 0.01, *** = *p* ≤ 0.001, **** = *p* ≤ 0.0001).

The CIA rat model is widely used to mimic the RA condition [22]. We used this model to investigate the effects of miR-4693-5p. Images of rat paws taken on the 34th day for all groups (Group 1 (HC), Group 2 (CIA), Group 3 (CIA + Negative Control (NC)), Group 4 (CIA + mimic), and Group 5 (CIA + MTX)) before scarification are shown (Figure 6). We observed that Groups 4 (CIA + mimic) and 5 (CIA + MTX) showed less redness and swelling compared with Groups 2 (CIA) and 3 (CIA + Negative Control (NC)) (Figure 6A). On days 0, 7, 14, 21, 28, and 34, the paw volume was measured using a plethysmometer to confirm the onset of the disease. After day 14, average paw volume decreased in Groups 4 and 5, whereas paw volume increased in Groups 2 and 3 (Figure 6B). The macroscopic arthritic score considerably decreased in Group 4 compared with Groups 3 and 2 (Figure 6C). After sacrificing the rats, the injected miRNA mimic was assessed in the rat synovium of Groups 3 and 4 by qRT-PCR to evaluate the overexpression of miR-4693-5p. We observed that Group 4 had higher levels of miR-4693-5p than Group 3, indicating an upregulated mimic level in rat synovium (Figure 6D). Levels of pro-inflammatory cytokines were also measured by ELISA in the rat plasma of all groups. Downregulation of pro-inflammatory cytokines (IL-1β, IL-6, and TNFα) was also revealed in rat plasma in Groups 4 and 5 compared with Groups 2 and 3 (Figure 6E). Therefore, our results suggest that miR-4693-5p might be responsible for the downregulation of pro-inflammatory cytokines and may have a protective effect in CIA rats by reducing paw volume, macroscopic arthritic score, and pro-inflammatory cytokine levels.

#### 2.11.2. Validation of the Anti-Inflammatory Effect of miR-4693-5p

To further validate the anti-inflammatory activity of miR-4693-5p, histological tests were performed on rat synovium by H&E staining (Figure 6F). The pink color represents cytoplasm, which correlates with the synovium’s inflammation. The purple color represents the number of nuclei present, and is used to determine the number of cells infiltrated into the given region to quantify inflammation [22]. The H&E scan analysis revealed that the group injected with miR-4693-5p (Group 4) and Group 5 (MTX) exhibited much less cell infiltration compared with Groups 3 and 2. This histological examination supports the idea that Group 4 selectively regulated pro-inflammatory cytokines through the NF-kB crosstalk, significantly reducing inflammation in vivo. These findings provide additional evidence of the therapeutic potential of miR-4693-5p in mitigating inflammation in a CIA rat model of RA.

#### 2.11.3. Immunohistochemical Analysis of HIF-1α and Survivin Expression

The expression of HIF1α in the synovial tissue in all groups was also measured to strengthen our findings further and was found to have less brown deposition in Group 4 compared with Groups 2 and 3, indicating less relative percentage area expression in Group 4 compared with Groups 2 and 3 (Figure 6G). Similarly, Survivin expression was reduced in Groups 4 and 5 compared with Groups 2 and 3 (Figure 6H). These findings confirm that miR-4693-5p suppressed the expression of HIF1α and Survivin, suggesting a protective role in impeding the progression of the disease. The downregulation of HIF1α and Survivin aligns with the observed anti-inflammatory effects of miR-4693-5p in the CIA rat model, further supporting its potential therapeutic significance in RA.

## 3. Discussion

RA is an autoimmune disease that causes joint damage and inflammation, but its exact cause remains unknown [1]. Studies show that overexpression of HIF1α in synovial joints leads to hyperplasia, immune cell infiltration, and resistance to apoptosis, aiding the development of RA pathogenesis [23], but the cause for overexpression of HIF1α in RA is unclear [6]. Given the potent regulatory role of miRNA in gene expression, several miRNAs, including miR-221, miR-448, miR-124, and miR-551b, have been identified in RA, playing regulatory roles in vital cellular processes like cell proliferation, angiogenesis, and inflammation, while their dysregulation contributes to RA pathogenesis [24].

It was reported that the miRNA expression of PBMCs might mimic conditions found in synovial tissue, making it easier to study in large samples [25]. Certain miRNAs such as miR146, miR145 [13], and miR21 [26] found in PBMCs are involved in RA pathogenesis. Our study is the first to report that miR-4693-5p was significantly downregulated in PBMCs of RA, suggesting miR-4693-5p may be a potential regulator in RA development. Therefore, we were interested in investigating the potential role of miR-4693-5p and attempted in silico studies followed by in vitro and in vivo studies. Next, our objective was to find the favorable target for miR-4693-5p, and our in silico target prediction results revealed that miR-4693-5p might target and bind to the 3′ UTR of HIF1α. Then, we validated the target in vitro, and the results confirmed that miR-4693-5p downregulates the expression of HIF1α in SW982 cells (Figure 1), demonstrating that miR-4693-5p is a potential regulator in RA development.

Pro-inflammatory cytokines initiate destructive processes in RA joints, leading to structural damage, reduced mobility, and functional decline, ultimately resulting in disability [27]. The report shows that proinflammatory cytokines can induce or stabilize HIF1α protein [3]. We explored the specific time relationship with HIF1α and TNFα (major cytokine involved in RA pathogenesis) and revealed that TNFα induces HIF1α expression in SW982 cells (24 and 48 h) in normoxic conditions (Figure 2D,E).

It is known that HIF1α regulates many proteins that are involved in carbohydrate metabolism, invasion, angiogenesis, cell proliferation, and apoptosis pathways [28]. While exploring more, PPI interaction results show that Survivin was an interacting protein of HIF1α, having the highest interaction score (Figure 3B) compared with the known proinflammatory cytokines (IL1β, TNFα, and IL6) involved in RA pathogenesis. It was reported that Survivin is transcriptionally regulated by HIF1α and also gets overexpressed in RA and inhibits intrinsic and extrinsic apoptotic pathways by interacting with caspase 3 and 9 to promote cell proliferation and hyperplasia, which aids disease progression [29]. Therefore, a study of the inhibition of Survivin expression in SW982 and RAFLS cells by HIF1α knockdown was conducted and validated (Figure 3C–E).

Our findings showed that miR-4693-5p upregulated the expression of proapoptotic proteins (Bax, CytC, and caspase 3/7) and downregulated the expression of antiapoptotic protein (Bcl2) (Figure 4) via HIF1α knockdown to promote apoptotic stimulation in SW982 and RAFLS cells, indicating that miR-4693-5p inhibits apoptosis resistance in RA to control disease progression [30].

The interaction between HIFs and NF-κB has been observed, and increased levels of both NF-κB and HIFs contribute to inflammatory responses by upregulating the expression of pro-inflammatory cytokines [20]. Moreover, NF-kB and HIF1α crosstalk in regulating the immune response in various immune cell types has also been observed, resulting in the stabilization of HIF1α in normoxia [20]. Recently, a report demonstrated that HIF1α increases the production of proinflammatory cytokines such as IL6, IL-8, TNFα, and IL1β [6]. Therefore, we checked the cytokine levels after HIF1α knockdown and found them to be downregulated (Figure 5A–C), demonstrating the anti-inflammatory activity of miR-4693-5p in RA (Figure 5) [25].

Hypoxia-inducible factor (HIF) accumulation and ROS formation occur simultaneously during hypoxia [31]. It was reported that downstream transcriptional targets of HIF could either activate or inhibit ROS formation, and an elevated level of ROS leads to HIF1α stabilization, resulting in increased HIF1α expression, and this stabilization governs ROS formation [21], which is also supported by our findings. We found that knockdown of HIF1α minimized the hypoxia conditions by suppressing ROS levels in TNFα-induced SW982 cells and RAFLS compared with the controls (Figure 5D,E), indicating that miR-4693-5p can reduce oxidative stress by reducing total cellular ROS levels.

Our findings were further validated by the CIA rat model. In total, downregulation of HIF1α and Survivin (Figure 6G,H) in the synovium of Group 4 (CIA + mimic) facilitated the decreased expression of pro-inflammatory cytokines compared with Groups 2 (CIA) and 3 (CIA + Negative Control (NC)) (Figure 6E). The current study provides a crucial foundation for understanding the potential role of miR-4693-5p as a promising therapeutic candidate and provides novel insights into the molecular mechanisms underlying RA pathogenesis.

## 4. Materials and Methods

### 4.1. Sample Collection

Blood and biopsy tissue samples were obtained from RA patients (*n* = 12) at the Orthopedic Department, All India Institute of Medical Sciences (AIIMS), New Delhi, India. Inclusion criteria: Patients were diagnosed with RA and met the revised 2010 American College of Rheumatology (ACR) and European League Against Rheumatism (EULAR) Rheumatism diagnostic criteria [32], and NSAIDs (nonsteroidal anti-inflammatory drugs, such as Ibuprofen, Diclofenac), DMARDs (Methotrexate), and surgery were advised as treatment options. Healthy Control (HC) blood samples (*n* = 12) were collected from individuals with no history of joint disease. Patients who were pregnant, alcoholic, or had other diseases such as diabetes, cardiovascular disease, or any other inflammatory diseases were excluded from the study. Detailed medical histories were recorded for all patients (Supplementary Table S1). Informed consent was obtained from all participants.

#### Blood Sample Processing

Plasma was separated from blood samples by centrifugation at 1300× *g* for 15 min (4 °C). The resulting plasma was processed, aliquoted, and stored at −80 °C for subsequent analysis [33].

### 4.2. miRNA Quantification in PBMCs

For quantification of miR-4693-5p, PBMCs were isolated using Histopaque (Sigma, St. Louis, MO, USA), and total RNA was extracted using Tri-Xtract Reagent (G-biosciences, St. Louis, MO, USA). cDNA synthesis was performed using a cDNA Synthesis Kit (G-biosciences) with stem-loop PCR. Expression analysis of miR-4693-5p was conducted using real-time PCR (Roche Light Cycler^®^ 480 Instrument-II, Mannheim, Germany). Data were quantitatively assessed using the 2^−ΔΔCT^ formula and normalized to U6 as an internal control [34]. Primer sequences are provided in Supplementary Table S2.

### 4.3. Target Prediction

Three miRNA target prediction databases (miRDB, RNA22, and TargetScan) were utilized to identify miR-4693-5p targets [14]. Common targets across databases were matched with RA-related genes from NCBI and DisGeNET. Selection criteria for favorable targets included a high total context score and 8mer binding site among targets, as well as the maximum number of proteins that interacted with the target [35].

### 4.4. In Vitro Studies

#### 4.4.1. Cell Culture (SW982)

SW982 cells, commonly used to study synovitis in RA, were purchased from the National Centre for Cell Science (NCCS). Cells were cultured in DMEM medium with antibiotics and 10% FBS at 37 °C with 5% CO_2_. Cells were then stimulated with Tumor Necrosis Factor-Alpha (TNFα) (10 ng/mL) [27].

#### 4.4.2. Primary Cell (RAFLS) Isolation

Synovium or synovial tissue was minced, digested with collagenase (0.5 mg/mL) (Sigma, St. Louis, MO, USA), plated, and incubated in DMEM containing 10% FBS at 37 °C with 5% CO_2_. Cells were used for the treatment after the second passage [29].

#### 4.4.3. Cell Transfection

For functional validity of miR-4693-5p, cells were transfected in a six-well plate until 60–70% confluency using Lipofectamine RNAiMAX Transfection Reagent (Invitrogen, Waltham, MA, USA) with 25 nM of miRNA Negative Control (NC), miR-4693-5p (mimic), and miRNA inhibitor (AntimiR or AM) (Invitrogen) in Opti-MEM media (Gibco, Waltham, MA, USA). After transfection (5 h), media were replaced with DMEM containing 10% FBS [10].

#### 4.4.4. Dual-Luciferase Reporter Assay

The TargetScan database was used to identify possible binding sites of miR-4693-5p to the 3′ UTR of HIF1α. Luciferase reporter vectors were constructed [36], and SW982 cells were co-transfected with miRNA and vectors. Five combinations were made: (1) Wild type (WT), (2) Mutant (Mut), (3) Wild type + Negative Control (WT + NC), (4) Wild type + mimic (WT + mimic), and (5) Mutant + mimic (MT + mimic). Luciferase activities were measured using a Tecan i-control infinite 200 Pro device (see details in Supplementary File).

#### 4.4.5. Real-Time PCR

After transfection, cells (SW982) were stimulated with TNFα (10 ng/mL). Total RNA was isolated and reverse-transcribed, and mRNA expression was determined by real-time PCR using human-specific primer sequences (Supplementary Table S2). The values were normalized by GAPDH and quantitatively evaluated using the 2^−ΔΔCT^ formula. Stem-loop primer was used to check the overexpression of miR-4693-5p in in vitro experiments using a specific primer and U6 as an internal control [27] (see details in Supplementary File).

#### 4.4.6. Western Blot (WB)

Cells were lysed in RIPA buffer, proteins were estimated using the BCA method [27], and 40 µg protein was separated by running SDS-PAGE. Then, proteins were transferred to the Nitrocellulose Membrane. The membrane was blocked (2 h) with 3% BSA, incubated overnight (4 °C) separately with the primary antibodies (Santacruz, Santa Cruz, CA, USA) Anti-human-HIF1α (1:500), Survivin (1:3000), CytC (1:2000), Bax (1:2000), and Bcl2 (1:2000), washed with 1XTBST, and incubated again (1 h) with HRP-conjugated secondary antibody (1:10,000, Jackson, MI, USA) at room temperature (RT). The blots were then developed with ECL and analyzed using the ChemiDoc system [34] (Bio-Rad Laboratories (Singapore) Pte. Ltd.) (see details in Supplementary File).

#### 4.4.7. Protein–Protein Interaction (PPI)

Interactive partners of HIF1α were identified using STRING and STITCH databases [35]. The downstream targets of HIF1α and their interactions with known pro-inflammatory cytokines were found to be related to RA pathogenesis.

#### 4.4.8. Caspase-Glo 3/7 Assay

The Caspase-Glo 3/7 Assay System (Promega, Madison, WI, USA) was used to evaluate caspase activity or apoptosis. After transfection, as described above, 100 µL of Caspase-Glo 3/7 reagent was added, followed by 3 h of incubation [37]. The luminescence was then measured using a Tecan i-control (see details in Supplementary File).

#### 4.4.9. Total ROS Estimation

Intracellular ROS formation was measured using DCFDA (Dichlorodihydrofluorescein diacetate). RAFLS and SW982 cells were transfected and induced with TNFα followed by 10 μM DCFDA (Invitrogen) addition into each well, incubation (30 min), and analysis of fluorescence intensity [38] (see details in Supplementary File).

### 4.5. In Vivo Studies

#### 4.5.1. Development of Collagen-Induced Arthritis (CIA) Rat Model

Female Wistar rats were obtained from the Indian Council of Medical Research–National Institute of Nutrition, Hyderabad, India. After two weeks of acclimatization, five groups (*n* = 4 rats in each group) were created: Healthy Control (HC) (Group 1), CIA (Group 2), Negative Control (NC)+ CIA (Group 3), CIA+ mimic (Group 4), and CIA+ MTX (Methotrexate, standard drug) (Group 5). CIA (not in the HC group) was caused by inducing 2 mg/mL collagen Type II from chicken (Sigma, USA) dissolved in 0.01 M acetic acid and combined (1:1) with complete adjuvant (Sigma, USA). miRNA (miR-4693-5p) (1 ng/gm of body weight or 200 ng/Rat) was administered to the rats intraperitoneally [39]. The rats were then sacrificed on day 34 [22,40] (see details in Supplementary File).

#### 4.5.2. Macroscopic Arthritis Score Evaluation

Arthritis severity was graded based on swelling, edema, and redness in all four paws of CIA rats using a 1 to 4 scale. A plethysmometer was used to calculate and quantify the swelling of the joints [41] (see details in Supplementary File).

#### 4.5.3. miRNA Isolation from Rat Synovium

Synovium was separated, and miRNA was extracted using a mirVana^TM^ miRNA Isolation Kit (Invitrogen). Expression of miR-4693-5p was determined by qRT-PCR [42] (see details in Supplementary File).

#### 4.5.4. Enzyme-Linked Immunosorbent Assay (ELISA)

Rat plasma was separated and added (100 µL) to the pre-coated ELISA plate, following the manufacturer’s guidelines. TNFα, IL(Interleukin)1β, and IL-6 cytokines were quantified using ELISA kits (ELK Biotechnology, Wuhan, China) [33].

#### 4.5.5. Hematoxylin and Eosin Staining (H & E)

For histological analysis, rat synovium slices (5 µm thick) were made using a microtome and stained with H&E, as per standard histological analysis protocol [40] (see details in Supplementary File).

#### 4.5.6. Immunohistochemistry (IHC)

For IHC analysis, rat synovium slices were immersed in 3% H_2_O_2_ solution (25 min) at room temperature (RT), blocked with 3% BSA (2 h), incubated overnight with HIF1α (1:500) and Survivin (1:500) antibodies (Santacruz) separately at 4 °C, and washed after incubation. The slices were then incubated (1 h) with the secondary antibody HRP conjugate, followed by incubation (10 min) with DAB (Diaminobenzidine) peroxidase substrate. Images of the slides were taken at a 10× magnification and examined using Image-J software (Version 1.54d) [22] (see details in Supplementary File).

### 4.6. Statistical Analysis

Graph Pad Prism (version 9.0) was used for statistical analysis, employing Student’s *t*-test, Mann–Whitney U, Analysis of variance (ANOVA), and Chi-square as appropriate (*p* < 0.05). Each experiment was repeated at least three times (see details in Supplementary File).

## 5. Conclusions

In our study, we observed a downregulation of miR-4693-5p in PBMCs of RA patients, indicating its role as a regulatory element of HIF1α. Additionally, we revealed that miR-4693-5p modulates apoptosis, inflammatory cytokine production, and cellular ROS response by targeting HIF1α and its downstream mediator, Survivin. These findings improve our understanding and provide novel insights into the molecular mechanisms driving the pathogenesis of RA. A limitation of the study was the inability to validate the expression of miR-4693-5p in large numbers of samples to consider miR-4693-5p as an effective therapeutic agent to ameliorate RA.

## Data Availability

For all original data and protocols, please contact Sagarika Biswas (sagarika.biswas@igib.res.in).

## Supplementary Materials

The following supporting information can be downloaded at: https://www.mdpi.com/article/10.3390/ncrna10020022/s1, Figure S1: Western blot analysis of HIF1α expression at different concentrations of hsa-miR-4693-5p ranging from 5–50 nm.; Figure S2: (A) Cloned confirmation of HIF1α 3’UTR in psiCHEK2 vector, (B) Site directed mutagenesis of seed region of HIF1α 3’UTR (mutated sequence shown in highlighted box); Figure S3: Significant overexpression of survivin confirmed in HC and RA plasma samples (*n* = 8 each) by ELISA; Table S1: The clinical demography characteristics of healthy control and patients with RA; Table S2: The amplification primers sequences of human-specific genes.
