# Supplementary material for: Functional Significance of miR-4693-5p in Targeting HIF1α and Its Link to Rheumatoid Arthritis Pathogenesis
Source: Noncoding RNA. 2024 Apr 10;10(2):22. doi: 10.3390/ncrna10020022 (PMC11053697; doi:10.3390/ncrna10020022)
Supplement: Supplementary file 1 [file ncrna-10-00022-s001.zip › ncrna-2927652-supplementary.pdf]

non-coding RNA

MDPI

Supplementary File

Functional Significance of miR-4693-5p in Targeting HIF1α and Its Link to Rheumatoid Arthritis Pathogenesis

Mohd Saquib [1,2], Prachi Agnihotri [1,2], Ashish Sarkar [1,2], Swati Malik [1,2], Sonia Mann [1], Debolina Chakraborty [1,2], Lovely Joshi [1,2], Rajesh Malhotra [3] and Sagarika Biswas [1,2,*]

1 Council of Scientific and Industrial Research (CSIR), Institute of Genomics and Integrative Biology, Mall Road, Delhi University Campus, Delhi 110007, India; saquibmkn@gmail.com (M.S.); prachiagnihotri21@gmail.com (P.A.); ashish2sarkar@gmail.com (A.S.); swatimalik5577@gmail.com (S.M.); mannsonia23@gmail.com (S.M.); debochak55@gmail.com (D.C.); lovelyabhilasha98@gmail.com (L.J.)
2 Academy of Scientific and Innovative Research (AcSIR), Ghaziabad 201002, India
3 All India Institute of Medical Science (AIIMS), Ansari Nagar, New Delhi 110029, India; rmalhotra62@gmail.com
* Correspondence: sagarika.biswas@igib.res.in; Tel.: +91-11-27667602; Fax: +91-11-27667471 or +91-98-18-004740

Materials and methods:

Enzyme-linked immunosorbent assay analysis (ELISA) (Survivin)

The indirect ELISA assay was conducted with plasma samples from $n = 8$ individuals (HC and RA), which were diluted at a ratio of 20µl plasma to 80µl coating buffer (consisting of 0.01M sodium carbonate and 0.035M sodium bicarbonate, pH = 9.6) and added to 96-well microtiter plates (Thermo Scientific, Nunc, USA). The plates were then incubated overnight at 4ºC. Following incubation, the wells were blocked with 1% bovine serum albumin (BSA, Sigma-Aldrich, USA) for 1 hour at room temperature. After blocking, the wells were washed with 0.1% Tween-20 in 1x PBS and then incubated for 2 hours with primary anti-Survivin antibodies (Santa Cruz, USA) diluted at 1:2000. Subsequently, the wells were treated with HRP-conjugated secondary antibodies (Jackson, USA) diluted at 1:1000 and incubated for 1 hour at room temperature. The reactions were initiated with ortho-phenylene diamine (1mg/ml) and halted by adding 3N H_2SO_4. Absorbance readings were taken at 495nm using an ELISA reader (Spectra Max Plus 384, Molecular Devices, USA).

Invitro-Studies:

Dual-luciferase Reporter Assay

TargetScan database (www.targetscan.org) was used to identify possible binding sites of hsa-miR-4693-5p to 3′ UTR of HIF1α (starting from 1193 to 1200 bp). To construct luciferase reporter vectors, 742bp 3′UTR sequence of human HIF1α was amplified using human cDNA, digested with XhoI and NotI restriction enzymes, and ligated in psiCHECK-2 vector (designated as Wild type). The mutant luciferase reporter constructs were generated by site-directed mutagenesis using overlap extension PCR (Supplementary Figure 2). SW982 cells were then seeded in a 6-well plate and co-transfected

(48 hours) with miRNA (25nM, 100 ng/well vector concentration). Five combinations were made: 1) wild type (WT), 2) Mutant (Mut), 3) wild type + Negative Control (WT + NC), 4) wild type + mimic (WT + mimic) and 5) Mutant + mimic (MT + mimic). Cells were then collected, and luciferase activities were measured using a Tecan i-control infinite 200 Pro device.

Real-Time PCR

Cells (SW982) were seeded in a six-well plate, transfected at 60-70% confluency, and stimulated (24 hours) with TNFα (10ng/ml) in a serum-free medium. Total RNA was isolated using Tri-Xtract RNA Isolation Reagent (G bioscience), and 1 μg of total RNA was reverse-transcribed using cDNA Synthesis Kit (G bioscience). The transcribed cDNA was combined with a 5x HOT FIREPol EvaGreen qPCR mix plus (ROX) master mix (SOLIS BIODYNE) and mRNA expression was determined by real-time PCR detection instrument (Roche Light Cycler II 480) using human-specific primer sequences (Supplementary Table 2). The values were normalized by GAPDH and quantitatively evaluated using $2^{-\Delta\Delta CT}$ formula. Stem-loop primer was used to check the expression of hsa-miR-4693-5p in *in-vitro* experiments using a specific primer, and U6 as an internal control.

Western Blot:

The SW982 cells were grown in DMEM containing 10% FBS in six wells plate, stimulated (24 hours) with TNFα (10ng/ml), and lysed in RIPA lysis buffer containing phosphatase and protease inhibitors. Protein (40 μg) estimated by the BCA method was loaded to run SDS-PAGE and transferred separated proteins onto nitrocellulose membrane (G Biosciences) using a semi-dry transfer unit (Bio-Rad Laboratories). The membrane was blocked (2 hours) with 3% BSA, incubated overnight (4°C) separately with primary antibodies (Santacruz); Anti-human-HIF1α (1:500),

Survivin (1:3000), CytC (1:2000), Bax (1:2000), and Bcl2 (1:2000), washed with 1×TBST, incubated again (1 hour) with HRP-conjugated secondary antibody (1:10000, Jackson, USA) at RT. The membrane was then washed, developed with ECL, and analyzed by using the ChemiDoc system (Bio-Rad Laboratories, Singapore) Pte. Ltd.)

Caspase-Glo 3/7 Assay:

The Caspase-Glo 3/7 Assay System (Promega) was used to evaluate the caspase activity or apoptosis. RAFLS cells were seeded in a 96-well plate, grown till 60-70% confluency, then transfected (48 hours) with NC, mimic, and AM (25nM) as described above, followed by 3 hours incubation after the addition of 100 μl of caspase-Glo 3/7 reagent. The luminescence was then measured using a Tecan i-control.

Total ROS estimation

Intracellular ROS formation was measured by fluorescence using 2′,7′-dichlorofluorescein diacetate (DCF-DA). RAFLS and SW982 cells were transfected with 25nM hsa-miR-4693-5p. SW982 cells were induced with TNFα (10ng/ml). Cells were washed with PBS, added 10μM DCFDA (Invitrogen) into each well, and incubated (30 minutes) at 37 °C. The DCFDA was removed and washed, and the fluorescence image was obtained by ZOE Fluorescent Cell Imager. The relative fluorescence intensity was analyzed by ImageJ software.

In-Vivo studies:

Development Collagen-induced arthritis (CIA) rat model

Female Wistar rats (60-80g) obtained from the ICMR-National Institute of Nutrition in Hyderabad, India. Rats were housed in an animal facility (25 ± 2°C,42% humidity) in a 12-hour day and dark

cycle, offered a conventional rodent chow diet and water ad-libitum. The work design was approved by the Institute's Animal Ethical Committee (IGIB/IAEC/3/3/Mar 2023). After two weeks of acclimatization, the animals were randomly divided into five groups ($n = 5$). The untreated group/ healthy control (HC) (Group 1), collagen-induced arthritis (CIA) (Group 2), negative control or non-targeted miRNA (NC+ CIA) (Group 3), hsa-miR-4693-5p mimic (CIA + mimic) (Group 4), and standard drug Methoxtrate group (1mg/kg) (CIA + MTX) (Group 5). For the induction of the CIA rats (Except HC group), collagen (Type II) from chicken (Sigma, USA) was dissolved (2mg/ml) in 0.01M acetic acid and combined (1:1) with complete adjuvant (Sigma, USA).

miRNA was administered at 1ng/gm of rat body weight. The collagen dosage was administered subcutaneously through the tail vein on the first and fourteenth days, miRNA dose was administered intraperitoneally (i.p) two days before the induction of CIA and on every third day until the 30th day. On the 34th day, rats were sacrificed using a combination of Thiopentone and Xylazine (3:1). Blood and synovium were collected for further evaluation.

Macroscopic arthritis score evaluation:

The arthritis score was determined by macroscopic examination of swelling, edema, and redness in all four paws of CIA rats. The severity was graded on a 1 to 4 scale: 1 indicates no obvious edema, swelling, or redness, 2 indicates moderately involved joints, 3 indicates highly involved joints with edema, redness, and swelling, and 4 indicates severely impacted joints with edema, swelling, and redness. A plethysmometer was used to calculate and quantify the swelling of the joints.

miRNA isolation from Rat Synovium:

Synovium (10 mg) was coarsely chopped, homogenized, solubilized in TRIZOL, and isolated miRNA using mirVana™ miRNA Isolation Kit (Invitrogen). Stem-loop primer was then used to make cDNA, followed by hsa-miR-4693-5p expression by qRT-PCR.

Hematoxylin and Eosin staining (H & E):

Rat synovium was sliced and fixed in 10% formalin, fixed in the paraffin block, and sliced (5µm thick) using a microtome. The slices were mounted on slides, deparaffinized, rehydrated with distilled water, and stained (1 minute) with hematoxylin. The slides were then dehydrated with ethanol, xylene washed, decolorized with 0.05% acid alcohol, rinsed, counter-stained with alcoholic Eosin (30 seconds), mounted coverslips, and viewed under a Nikon microscope. images of the slides at 10X magnification were taken, and Image-J software was used to analyze the images.

Immunohistochemistry (IHC):

The tissue slices were deparaffinized with xylene, hydrated with ethanol, and then antigen retrieval was performed in the microwave for 23 minutes. Slices were cooled and immersed in 3% H_2O_2 solution (25 minutes) at RT, blocked with 3% BSA (2 hours), incubated overnight with HIF1α (1:500) and survivin (1:500) antibodies separately at 4°C. The slices were then incubated (1 hour) with the secondary antibody MedaViewTM one-step Polyer-HRP anti-mouse and then incubated for 10 minutes with a DAB peroxidase substrate. The samples were stained with haematoxylin for 3 minutes, submerged in ethanol and xylene, fixed with neutral resin, and examined under Nikon ECLIPSE 90i microscope. Slide images were taken at a 10X magnification, and Image-J software was used to examine the image.

Statistical Analysis:

Statistical analysis was conducted using GraphPad Prism (version 9.0) (https://www.graphpad.com/). The comparison of data from two or more groups involved employing the student's t-test, Analysis of Variance (ANOVA), and Chi-square for categorical data, with significance determined at $p < 0.05$. Each experiment was repeated at least three times.

Supplementary Figures Legends

Supplementary Figure S1: Western blot analysis of HIF1*α* expression at different concentrations of hsa-miR-4693-5p ranging from 5-50nm.

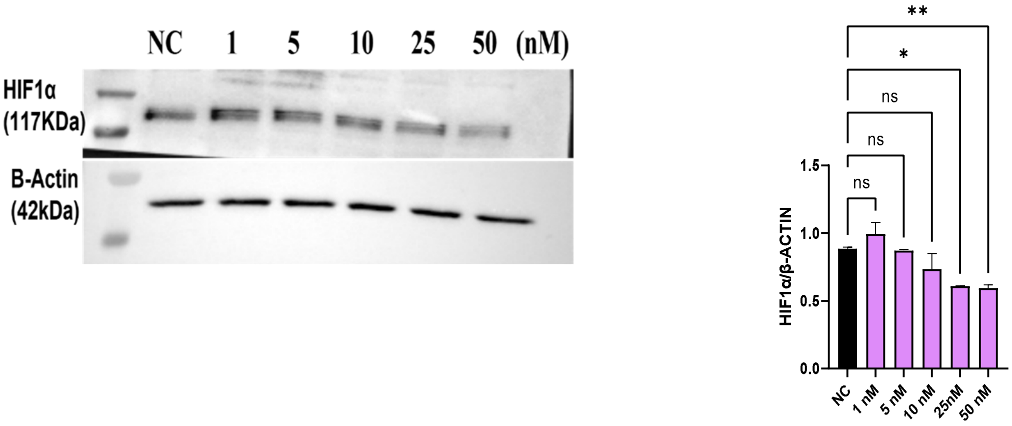

Supplementary Figure S2: **A**) Cloned confirmation of HIF1*α* 3′UTR in psiCHEK2 vector **B)** Site directed mutagenesis of seed region of HIF1*α* 3′UTR (mutated sequence shown in highlighted box)

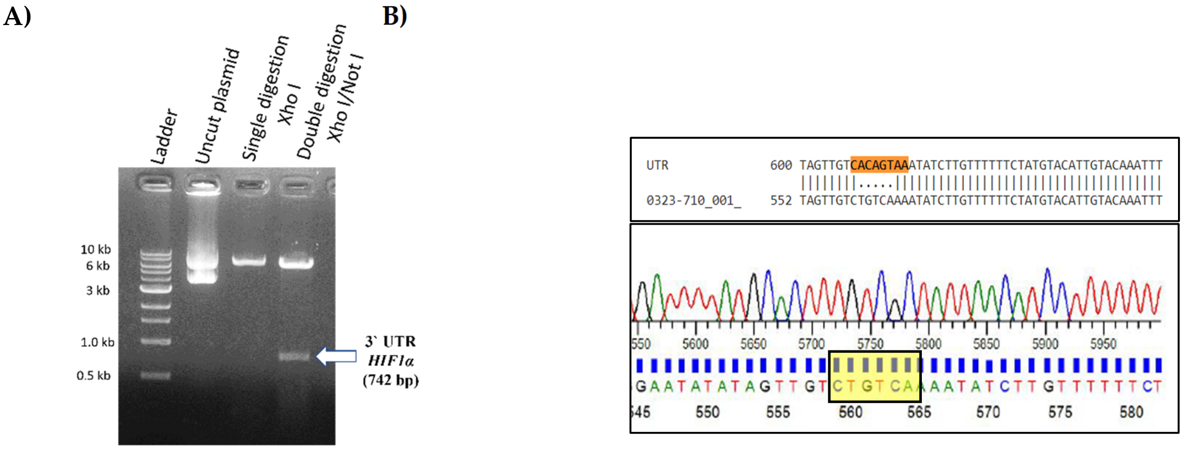

Supplementary Figure S3: Significant overexpression of survivin confirmed in HC and RA plasma samples (*n* = 8 each) by ELISA.

Survivin Elisa
plasma samples (*n* = 8 each)

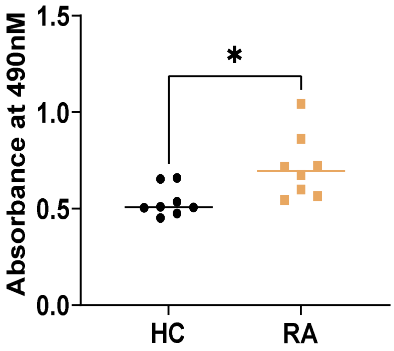

Supplementary Table S1: The clinical demography characteristics of healthy control and patients with RA.

**S. No.**	**Patient's characteristics**	**RA (*n* = 12)**	**HC (*n* = 12)**
1	Age (yrs.)	50 ± 5	45 ± 10
2	Sex (Female, Male)	F (6), M (6)	F (6), M (6)
3	ESR (mm/hr)	35 ± 5	-
4	RF (+ve/-ve)	+ve	- ve
5	CRP (mg/L)	80 ±15	10 ± 5
6	Tender Joint	20 ± 6	-
7	Swollen joints	10 ± 4	-
8	DAS-28 score	6 ± 0.5	-

9	Disease duration yrs.	10 ± 5	-
10	Medication (Yes/No)	Yes	No

*RA: Rheumatoid arthritis; HC: Healthy control; OA Osteoarthritis; ESR: Erythrocyte sedimentation rate; RF: Rheumatoid factor; CRP: C-reactive protein; DAS-28: Disease Activity Score-28. The values are expressed as Mean ±SD

Supplementary Table S2. The amplification primers sequences of human-specific genes.

Sr.	Gene Name	Primer Sequence Forward	Primer Sequence Reverse
1	HIF1α	5′-GAAAGCGCAAGTCCTCAAAG-3′	5′-TGGGTAGGAGATGGAGATGC-3′
2	hsa-miR-4693-5p	5′-CGGCGGCATACTGTGAATTTC-3′	5-GTCGTATCCAGTGCAGGGTCC-3′
3	hsa-miR-4693-5p Stemloop primer	5′- GTC GTA TCC AGT GCA GGG TCC GAG GTA TTC GCA CTG GAT ACG ACTGTGACAG -3′	
4	U6	5′-GCGGCGGCGCAAGGATGACACG-3′	5′-ATCCAGTGCAGGGTCCGAGG-3′

5	IL-6	5′-GGTACATCCTCGACGGCATCT-3′	5′-GTGCCTCTTTGCTGCTTTCAC-3′
6	TNF-α	5′-CCCCAGGGACCTCTCTCTAATC-3′	5′-GGTTTGCTACAACATGGGCTACA-3′
7	IL-1β	5′-AAACAGATGAAGTGCTCCTTCCAGG- 3′	5′-TGGAGAACACCACTTGTTGCTCCA- 3′
8	GAPDH	5′-GAAGGTGAAGGTCGGAGTC-3′	5′-GAAGATGGTGATGGGATTTC-3′
